# Interest in Insects as Food and Feed: It Does Not Wane in the Public Domain

**DOI:** 10.3390/foods11203184

**Published:** 2022-10-12

**Authors:** Victor Benno Meyer-Rochow, Chuleui Jung

**Affiliations:** 1 Agricultural Science and Technology Research Institute, Andong National University, Andong 36729, Korea; 2 Department of Ecology and Genetics, Oulu University, SF-90140 Oulu, Finland; 3 Department of Plant Medicals, Andong National University, Andong 36729, Korea

This Special Issue of *Foods* represents Volume 2 of the topic “Edible Insects as Innovative Foods: Nutritional, Functional and Acceptability Assessments”. Although some of the 20 contributions in this volume deal with hitherto unreported food insects and some explore the effects that a diet containing insects or insect products has on the gut microbiota of the consumer, whether human or non-human, most of the articles deal with improvements related to processing and rearing food insects. Food safety questions are not ignored and questions of the acceptability of insect containing food stuffs are not either. What affects the palatability and the chemical composition of farmed insects most, is explored and how protein and mineral levels in edible insects can be increased is dealt with in several articles. Finally, the plea is made not to focus only on the reasons why some people reject insects as food, but to provide convincing reasons for the advantages to health and the environment that a greater use of insects as food and feed would present.

**Figure 1:**
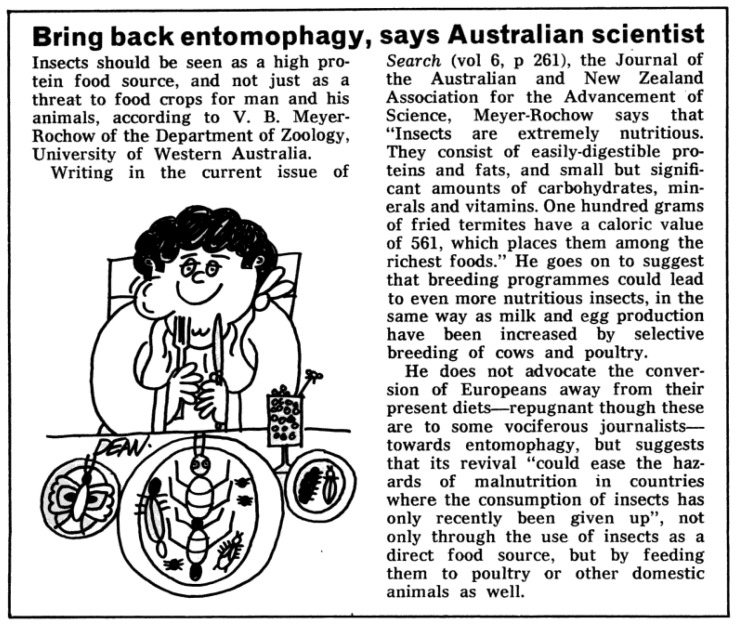
One of many cartoons that appeared in newspapers and magazines [12] after it had been suggested that insects could ease the problem of food shortages.

It is exactly 47 years ago that for the first time it had been suggested by Meyer-Rochow that the use of insects as food and feed could ease the problem of global nutritional shortfalls and that WHO and FAO should be encouraged to support the use of insects as food [1]. Ridiculed at first, and not taken seriously even by some science magazines and often accompanied by funny cartoons (Figure 1), the idea gradually gained momentum and the suggestion began to be dealt with at conferences when the topic of Food Security was discussed. Recognition by the FAO finally came when a renewed call for support was published by Van Huis et al. [2] and the need to increase global food production to feed the increasing world population could no longer be overlooked.

The extraordinary increase in publications dealing with edible insects and entomophagy from 1900 to 2015 was documented in 2015 by Evans et al. [3] and Müller et al. [4]. Since then, hundreds of additional papers, too many to list individually, covering a huge range of issues all related to the use of insects as food and feed, have appeared. Yet, there seems to be no sign of a waning interest in insects that are being promoted as a ‘novel food for humans’ (which is actually wrong as insects represent a very ancient kind of food for humans) [5]. In fact, products that contain insect material are now becoming increasingly available and insects reared to be fed to livestock and poultry and to be used in fish culture have become popular alternatives to conventional feed [6,7,8,9,10].

Volume one of our Special Issue on “Edible Insects as Innovative Foods: Nutritional, Functional and Acceptability Assessments” appeared in 2020 and contained 20 articles [11]. Volume two also contains 20 articles, but the emphasis now seems to have shifted somewhat and this time the Special Issue includes more papers related to the effects of rearing conditions on the composition and the nutritive status of commercially reared insets and how to best utilize these insects. Authors from 13 different countries were involved in the articles that make up volume 2 and while it is obvious that food insects can no longer be ignored as a food or feed item, a number of issues remain to be explored.

Grinding pupae of honeybee drones and using that powder as an ingredient in puffed-rice snacks has been carried out by Woo-Hee Cho in Korea [13]. To what extent different heating and drying conditions affected the puffed-rice snack product and how the chemical composition of the latter differed from the control were subjects of the study. The product enriched by pupal drone bee powder showed a higher content of proteins, fats, amino acids and fatty acids and it was concluded that the developed product could be consumed as a nutritional snack, although the quality characteristics could still be improved through optimal processing.

Effects of various de-fattening methods on the physicochemical properties of proteins extracted from *Hermetia illucens* larvae were studies by Tae-Kyung Kim who found that the total essential amino acid contents were higher with cold pressure protein extraction than with other treatments [14]. Cold pressure-defatted protein showed the highest emulsifying capacity while water extracted protein showed the lowest emulsifying capacity. Although organic solvents may be efficient for defatting proteins extracted from insects, they have detrimental effects on the human body. Moreover, the organic solvent extraction method requires a considerable amount of time for lipid extraction. Using cold pressure protein extraction on edible insect proteins is eco-friendly and economical due to the reduced degreasing time and its potential industrial applications.

Maiyo et al. [15] investigated whether the nutritional quality of four novel porridge products blended with edible cricket (*Scapsipedus icipe*) meal is improved to an extent that it can be recommended to reduce incidences of malnutrition in low and middle-income countries. Porridge enriched with the meal of the newly discovered cricket *Scapsipedus icipe* had significantly higher protein (2-fold), crude fat (3.4–4-fold), and energy (1.1–1.2-fold) levels than the commercial porridge flour. Fermented cereal porridge fortified with cricket meal had all three types of omega-3 fatty acids and germinated cereal porridge with cricket meal had a significantly higher iron content (19.5 mg/100 g). In conclusion, to fortify porridge products with cricket meal is to be recommended.

In a study by Vanqa et al. [16] edible insect flours from *Gonimbrasia belina* (Mashonzha), *Hermetia illucens* (black soldier fly larvae) and *Macrotermes subhylanus* (Madzhulu) were assessed in terms of proximal, physicochemical, techno-functional and antioxidant properties. Crude protein of the edible insect flours varied between 34.90–52.74% but there were no significant differences (*p* > 0.05) in foam capacity and foam stability of all three edible insect flours. The findings revealed that the flours of the three edible insects are a good source of antioxidants and can be used as an alternative protein source and a potential novel food additive due to their techno-functional qualities.

Investigating the nutritional, techno-functional and structural properties of Black Soldier Fly (*Hermetia illucens*) larval flours (BSFL) and protein concentrates has been the subject of the paper by Mshayisa et al. [17]. The highest protein content (73.35%) was obtained under alkaline and acid precipitation extraction (BSFL-PC1). The sum of essential amino acids significantly increased (*p* < 0.05) from 24.98% to 38.20% due to the defatting process during extraction. The protein extraction method influenced the structural properties of the protein concentrates and in conclusion it can be stated that BSFL flour fractions and protein concentrates show promise as novel functional ingredients for use in food applications.

Gan et al. [18] explored effects of different processing and packaging methods on lipid oxidation of deep-fried crickets (*Gryllus bimaculatus*) during storage. The composition of fatty acids changed and the content of FFA, PV, and TBAR values also increased with the extension of storage time, indicating that the lipid oxidation dominated by oxidation of unsaturated fatty acids could occur in deep fried *Gryllus bimaculatus* during storage. The study revealed that dibutyl hydroxyl toluene (BHT) was the most effective antioxidant.

An experiment by Selaledi et al. [19] was conducted to examine the effects of larval yellow mealworm meal (*Tenebrio molitor*) added to the diets of indigenous chicken. Boschveld chickens were randomly divided into four categories. Controls were given only soybean meal (SBM) and yellow mealworm with percentage levels of 5, 10 and 15 (TM5, TM10 and TM15, respectively) were used in the experiment. The authors demonstrated that dietary *Tenebrio molitor* in growing Boschveld chickens could be regarded as a promising added protein source, improving quality rearing of Boschveld chickens.

A more traditional investigation was that by Dürr and Ratompoarison [20], who provided evidence for the importance of food insects in the local diet of rural communities in the central highlands of Madagascar. The investigation showed that the insects contributed significantly to the animal protein uptake of the local population, especially in the humid season, when other protein sources were scarce. The authors then discussed how traditionally appreciated insects could be promoted in food-insecure rural areas and how entomophagy could support food and nutrition security in a growing population.

Ying Su [21] report that the larvae of the sphingid moth *Clanis bilineata tsingtauica* Mell 1922 are commonly used as food for humans in the eastern part of China. This insect was found to be high in nutrients, particularly in the epidermis where protein total was 71.82%. In this case, 16 different amino acids were quantified and the ratio of essential to nonessential amino acids in the epidermis and meat was 68.14% and 59.27%, respectively. Thestudy confirmed that *C. bilineata tsingtauica* was a highly nutritious food source for human consumption, and the results provide a basis for further uses and industrialization of this edible insect.

Storing edible insects and increasing their shelf lives are important issues. However, so are storage conditions for mass reared edible species, which is why Zhu et al. [22] set out to determine the optimal temperature under which eggs of the sphingid *Clanis bilineata tsingtauica* Mell, 1922 should be kept. Considering various combinations, the authors found that optimal egg hatching occurred if eggs were stored at 15 °C for 11 days, and then held at 15–20 °C under dark conditions. The conditions described allow for easier mass rearing of *C. bilineata tsingtauica* by providing a stable supply of eggs throughout the year.

Researchers from Iceland headed by Rune Thrastardottir et al. [23] presented an overview of the most popular insects farmed in Europe, namely the yellow mealworm, *Tenebrio molitor* and the black soldier fly (BSF) *Hermetia illucens*, focusing on the main obstacles and risks associated with maintenance. The results showed that the insect farming industry was increasing in Europe, and that the success of the frontrunners of the farmed insect industry was based on large investments in technology, automation and economy. However, more information still had to be forthcoming regarding risks posed by edible insects in terms of food safety. 

Food safety and risk assessment was also the topic of the study by Anja et al. [24]. These researchers investigated samples of edible insect species for the presence of antimicrobial-resistant and Shiga toxin-producing *Escherichia coli* (STEC). The presence of genes associated with antimicrobial resistance or virulence, including *stx1*, *stx2,* and *eae*, was investigated by PCR. The study showed that STEC can be present in edible insects, representing a potential health hazard. By contrast, the low resistance rate among the isolates indicated a low risk for the transmission of antimicrobial-resistant *E. coli* to consumers.

In the study by Kipkoech et al. [25] cricket chitin was deacetylated to chitosan and the latter was added in various concentrations to *Salmonella/Shigella* growth media. Growth of the probiotic bacteria was monitored on chitosan-supplemented media after 6, 12, 24, and 48 h upon incubation at 37 °C. The good news is that all chitosan concentrations significantly increased the populations of probiotic bacteria and decreased the populations of pathogenic bacteria. This study suggests that cricket-derived chitosan can function as a prebiotic, with an ability to eliminate pathogenic bacteria in the presence of probiotic bacteria.

That a partial substitution of meat with insects (*Alphitobius diaperinus*) in a diet for rats can change the gut microbiome was shown by Lanng et al. [26]. These researchers following a four-week dietary intervention in a healthy rat model could show that metabolomics analyses revealed a larger escape of protein residues into the colon and a different microbial metabolization pattern of aromatic amino acids when pork was partly substituted by insects. It could be shown that the introduction of insects in a common Western omnivorous diet altered the gut microbiome diversity with consequences for the endogenous metabolism.

The food an insect consumes can affect the insect’s chemical constitution and when leftovers of food originally for humans are the given to insects, this can affect the insects in many ways. The Black Soldier Fly (BSF) is able to thrive on a wide range of leftovers and especially vegetable processing industries generate huge amounts of by-products that can be used to rear BSF. Significant lower protein contents were detected by Fuso et al. [27] in BSF grown on fruit by-products, while higher contents were observed when autumnal leftovers were employed. Lysine, valine and leucine were amino acids most affected by the diet, but essential amino acids generally satisfied the Food and Agricultural Organization (FAO) requirements for human nutrition with the exception of lysine.

Placentino et al. [28] focused in their study on the diet of professional athletes and tried to discern what motivated professional athletes to accept an energy protein bar fortified with cricket flour. A second aim was how information on the benefits of edible insects impacted their acceptance as food by the athletes. The researchers’ results showed that the protein content and the curiosity about texture were the main drivers to taste the cricket energy bar; but the feeling of disgust justified the rejection of tasting insects. Although male athletes were more likely than females to endorse the product that contained cricket flour, the authors point out that a relatively small sample was involved, and therefore advise caution not to draw hasty conclusions. 

The aim of the study by Son et al. [29] was to investigate the carbohydrate content and composition of mealworms and to determine the amount of chitin. The crude carbohydrate content of mealworms was 11.5%, but the total soluble sugar content was only 30% of the total carbohydrate content and fructose was identified as the most abundant free sugar in mealworms. With a yield of 4.7%, chitin derivatives were the key components of mealworm carbohydrate. Although similar to crustacean chitin, mealworm chitin exhibited a significantly softer texture than crustacean chitin and showed superior anti-inflammatory effects.

Hornets have become increasingly popular as a food item because of their nutritional value and abundance. This is why Ghosh et al. [30] analyzed the nutrient compositions of the edible broods of *Vespa velutina*, *V. mandarinia*, and *V. basalis*. Farmed *V. velutina* and *V. mandarinia* were found to have similar protein contents, i.e., total amino acids, but *V. basalis* contained less. In all three species: leucine followed by tyrosine and lysine were predominant among the essential and glutamic acid among the non-essential amino acids. Polyunsaturated fatty acids were dominant in *V. mandarinia* and *V. basalis*, but saturated fatty acids were most abundant in *V. velutina.* It is concluded that the high content, especially of micro minerals such as iron, zinc, and the high K/Na ratio in hornets could help mitigate mineral deficiencies among those with inadequate nutrition.

Aquatic insects, although consumed by many, feature less often in research on human entomophagy. This is why the paper by Min Zhao et al. [31] is important. The authors reviewed what is known about edible aquatic insects and pointed out that the vast majority of the latter, in contrast to the phytophagous terrestrial edible species, are carnivorous. There are differences in, for example, fat, fatty acids and mineral content between terrestrial and aquatic insects and regarding food safety, it is advisable not to consume large quantities of wild aquatic species as they could contain higher amounts of heavy metals, pest residues and uric acid than phytophagous species.

A review covering differences in the chemical composition and nutrient quality (and thereby acceptability of edible insects) by Meyer-Rochow et al. [32], emphasized that multiple reasons can lead to such differences. Choice of the insect species, collection site, processing method, insect life stage, rearing technology and insect feed can affect an individual’s palatability and acceptance. According to the authors, the review can assist the food insect industry to select the most suitable species as well as processing methods for insect-based food products.

In the past a great deal of effort has been spent on recording which kinds of insects were consumed where and by whom [5,33,34,35], but as of late the emphasis has been shifting to analyzing the chemical composition of the edible species and, as this Special Issue has demonstrated, on determining optimal methods to rear and process the insects. Obviously, there are still communities and areas which have not been visited and interviewed, so that a complete picture where and how people eat which kinds of insect is still incomplete. Furthermore, as some articles in this Special Issue have shown, we still do not yet have information on the chemical contents of all known edible insects and do not yet know what affects their composition. However, the majority of the papers in this Special Issue deal with assessing quality questions of the insect-containing product and that presently seems to be one of the main areas of interest.

Food safety issues, of course, came up as well and, no doubt, will continue to be one of the foci of research in the future, similar to how insect chitin will be with its many possible applications in the health sector [36]. Insects have been used therapeutically since time immemorial and microbiological tests have been used to demonstrate the effectiveness of certain insect-derived substances to fight infections and other illnesses [37,38,39,40]. What to some extent seems surprising, is that only a handful of insect species, i.e., primarily crickets, black soldier fly and mealworms, receive the bulk of attention. Perhaps they do represent species that are easiest to culture as they accept a variety of food stuffs, feature short generation times, are unproblematic in their requirements, can be used in multiple ways and provide acceptable returns of the investment put into their maintenance. However, there may well be other species whose potential has not yet been fully realized, e.g., drone bees, hornets and wasps come to mind.

As we have already pointed out in the first volume of the Special Issue on “Edible Insects as Innovative Foods: Nutritional, Functional and Acceptability Assessments”, insects have many advantages over conventional meat-supplying species. The former requires less space than the latter, their rate of reproduction is considerably higher, a much greater percentage of an insect’s body mass can be used as food and the so-called “carbon footprint” of cultured insects is considered to be much lower than that of conventional animals used as food [41,42,43], which is due largely to the insects’ much lower water and food needs and their significantly higher food conversion rate. Thus, there is less “waste” generated by farmed insects. What needs to be entered into the equation, however, is the need to keep farmed insects warm and pest free, but this is not likely to change the conclusion that overall farming insects can be achieved with fewer negative side effects than rearing big mammals and poultry.

As people in countries originally famous for entomophagy increasingly abandon insects as a traditional food and begin to reject insect-containing food [44], there is a need to promote edible insects and food items that contain insect products. It is not only important to learn why people reject insects as food [45,46,47,48], it is more important to convince people to accept farmed edible insects as the latter are wholesome and nutritionally valuable. Education, clever marketing, turning to traditional recipes, tapping into the trend of buying healthy food and highlighting nutritional and medical benefits, can all help consumers to consider buying insect-containing food. Curiosity should be encouraged. In addition, as with the first volume of this Special Issue topic [49], we end this paper with the suggestion “Mealworms and spaghetti is food that makes you happy” and advise the readers to “Forget about the pork and put a cricket on your fork!”.
